# The Role of Ceramide and Sphingosine-1-Phosphate in Alzheimer’s Disease and Other Neurodegenerative Disorders

**DOI:** 10.1007/s12035-018-1448-3

**Published:** 2019-01-05

**Authors:** Kinga Czubowicz, Henryk Jęśko, Przemysław Wencel, Walter J. Lukiw, Robert P. Strosznajder

**Affiliations:** 1 Laboratory of Preclinical Research and Environmental Agents, Department of Neurosurgery, M. Mossakowski Medical Research Centre, Polish Academy of Sciences, 5 Pawinskiego St., PL-02106 Warsaw, Poland; 2 Department of Cellular Signalling, Mossakowski Medical Research Centre, Polish Academy of Sciences, 5 Pawinskiego St., PL-02106 Warsaw, Poland; 3 LSU Neuroscience Center and Departments of Neurology and Ophthalmology, Louisiana State University School of Medicine, New Orleans, LA 70112 USA

**Keywords:** Alzheimer’s disease, Ceramide, Huntington’s disease, microRNA, Parkinson’s disease, Sphingosine-1-phosphate

## Abstract

Bioactive sphingolipids—ceramide, sphingosine, and their respective 1-phosphates (C1P and S1P)—are signaling molecules serving as intracellular second messengers. Moreover, S1P acts through G protein-coupled receptors in the plasma membrane. Accumulating evidence points to sphingolipids' engagement in brain aging and in neurodegenerative disorders such as Alzheimer’s, Parkinson’s, and Huntington’s diseases and amyotrophic lateral sclerosis. Metabolic alterations observed in the course of neurodegeneration favor ceramide-dependent pro-apoptotic signaling, while the levels of the neuroprotective S1P are reduced. These trends are observed early in the diseases’ development, suggesting causal relationship. Mechanistic evidence has shown links between altered ceramide/S1P rheostat and the production, secretion, and aggregation of amyloid β/α-synuclein as well as signaling pathways of critical importance for the pathomechanism of protein conformation diseases. Sphingolipids influence multiple aspects of Akt/protein kinase B signaling, a pathway that regulates metabolism, stress response, and Bcl-2 family proteins. The cross-talk between sphingolipids and transcription factors including NF-κB, FOXOs, and AP-1 may be also important for immune regulation and cell survival/death. Sphingolipids regulate exosomes and other secretion mechanisms that can contribute to either the spread of neurotoxic proteins between brain cells, or their clearance. Recent discoveries also suggest the importance of intracellular and exosomal pools of small regulatory RNAs in the creation of disturbed signaling environment in the diseased brain. The identified interactions of bioactive sphingolipids urge for their evaluation as potential therapeutic targets. Moreover, the early disturbances in sphingolipid metabolism may deliver easily accessible biomarkers of neurodegenerative disorders.

## Introduction

Aging, which itself influences the central nervous system (CNS) in a relatively subtle manner, creates vulnerable background for the development of devastating disorders. Two of the most widespread neurodegenerative diseases are Alzheimer’s (AD) and Parkinson’s (PD). Dementia affects estimated 47 million people worldwide [1], placing enormous burden on the affected individuals, their families, societies, and healthcare systems. AD is the most common neurodegenerative disorder, responsible for up to 70% of dementia cases [1]. This disease is characterized by the presence of aggregates of pathologically misfolded proteins, including the extracellular senile plaques built mainly of amyloid β (Aβ), a product of proteolytic cleavage of the transmembrane Aβ precursor protein (AβPP) by β- and γ-secretase [2]. Neurons in AD also display cytoskeletal abnormalities that are linked to hyperphosphorylation and aggregation of the microtubule-associated tau protein into intracellular neurofibrillary tangles [3].

Parkinson’s disease is the most frequently occurring movement disease and the second most widespread neurodegenerative disorder after AD [4]. It is estimated that PD affects up to 1% of people over the age of 60 and up to ca. 4% over 85 [5]. PD is characterized by subcortical neurodegeneration, including the characteristic loss of dopaminergic phenotype neurons in substantia nigra pars compacta, loss of dopaminergic striatum innervation, and histopathological aberrations in the form of α-synuclein (ASN)-containing intracellular Lewy bodies (LB)/Lewy neurites (LN) [4]. Disturbances in other neurotransmitter systems (serotoninergic, noradrenergic, and cholinergic) are increasingly being recognized along non-motor symptoms, which—in later stages—may include dementia [6]. Like AD, Parkinsonian neurodegeneration progresses in a stealthy manner, and when clear symptoms appear the dopaminergic neuron population is already decimated [4].

### Bioactive Sphingolipids Biosynthesis

Once merely considered structural compounds, bioactive sphingolipids are increasingly implicated as signaling molecules in the brain, and play important roles in aging, neurodegenerative disorders, and the accompanying immune deregulation [7]. Ceramide, ceramide-1-phosphate (C1P), sphingosine, and sphingosine-1-phosphate (S1P) are the best described bioactive sphingolipids regulating stress resistance, proliferation, differentiation, and mature phenotypes of nervous system cells [8, 9]. Sphingolipids have multiple ancillary roles in the regulation of cell growth, death, senescence, adhesion, migration, inflammation, angiogenesis, and intracellular trafficking in the CNS [10, 11]. The *sphingolipid rheostat* model ascribed these compounds clearly opposite roles in cellular survival signaling: ceramide as a cell death activator, while C1P and S1P promoted survival. The fact that single phosphorylation reaction turns ceramide and sphingosine into their antagonistic counterparts stresses the significance of the precise, successful control of sphingolipid metabolism enzymes [8, 12]. Although the pro- versus anti-survival roles have blurred somewhat in recent years [13], the critical roles of sphingolipid signaling in nervous system function have been confirmed by effects of mutations in their biosynthesis and receptor genes [14–24]. The importance of bioactive sphingolipids is also stressed by the accumulating evidence about their involvement in aging and neurodegenerative disorders [8, 25–29].

The rate of ceramide biosynthesis is controlled by the first step of the pathway’s de novo branch, which is catalyzed by serine palmitoyltransferase (SPT). SPT product dihydrosphingosine is then metabolized by ceramide synthase (CERS) to dihydroceramide, which is subsequently converted by dihydroceramide desaturase (DES or DEGS) to ceramide. Ceramide may be metabolized into sphingomyelin by sphingomyelin synthase (SMS, or SGMS); the reverse reaction (the *sphingomyelinase pathway*) catalyzed by sphingomyelinases (SMases, SMPDs) is another major ceramide source. Ceramide also serves as a precursor for the production of sphingosine by ceramidases. CERS can perform the opposite reaction, which is third ceramide source, termed the *salvage pathway*. Sphingosine kinases (SPHK1, SPHK2) phosphorylate sphingosine into S1P in a highly regulated fashion in various cellular compartments; dephosphorylation is carried out by S1P phosphatases (SGPP1 and SGPP2), while S1P can be also hydrolyzed irreversibly into ethanolamine phosphate and hexadecenal by S1P lyase (SGPL) [9]. Activity of the enzyme glucocerebrosidase (GBA) can be another ceramide source [30] with significant links to Parkinson’s disease (see Pt. 'The role of bioactive sphingolipids in Parkinson’s disease').

### S1P and Ceramide in Neuronal Survival and Death Signaling

**Fig. 1:**
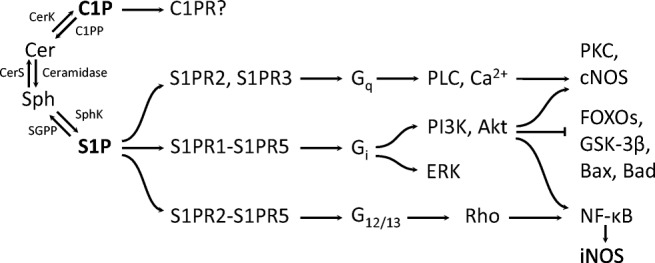
**Signaling pathways triggered by the cell surface receptors for S1P (S1PRs) and C1P (C1PR).** S1P through its G protein-binding receptors modulates pathways known for their engagement in the regulation of cellular metabolism oxidative/nitrosative stress and death/survival. The depicted examples of S1PR-activated signaling pathways are far from exhausting the spectrum of observed interactions (e.g., S1PR2 inhibits Akt while S1PR3 activates it; iNOS is typically induced by NF-κB, but can also undergo S1P-/C1P-dependent suppression via p38 MAPK [8, 33]). In contrast, C1PR, the cell surface receptor for ceramide-1-phosphate, remains poorly characterized and it is generally not known what part of C1P effects it mediates. According to [34], modified

Bioactive sphingolipids employ several mechanisms to exert their influence on intra- and extracellular signaling pathways. Plasma membrane is one of main cellular regions of SPHK activation; S1P and probably C1P can bind cell surface receptors [31, 32]. S1P receptors (S1PR1 to S1PR5) belong to the Edg family and bind G_q_, G_i_, G_12/13_, and Rho proteins [31]. While data on the postulated C1P receptor is scarce, all S1PR1 to -5 bind G_i_, S1PR2 and -3 bind G_q_ and all but S1PR1 signal through G_12/13_ (Fig. 1). Through either S1PR-binding G proteins or through more direct interactions with intracellular enzymes, sphingolipids also modulate signaling events such as cAMP (cyclic adenosine monophosphate), MAPK (mitogen-activated protein kinase), PKC (protein kinase C), PLD (phospholipase D), and PI3 kinase–Akt–PKB (protein kinase B) [35, 36]. The PI3K–Akt pathway is a crucial integrator of metabolic and stress signals engaged in a plethora of physiological and pathological processes ranging from energy metabolism through aging to age-related diseases [8]. Sphingolipids are also structural components of lipid bilayers. Changes in their local concentrations modify membrane properties further modulating signaling events that take part in these membranes.

Ceramide controls not only multiple cell death mechanisms but also cellular senescence, differentiation, and aspects of arborization in neurons [37, 38]. Sphingosine also seems to be engaged in cell death modulation [39]. C1P has been shown to stimulate cellular survival, growth, and may counteract ceramide signaling also through downregulation of acid sphingomyelinase and serine palmitoyltransferase activities [40, 41]. S1P regulates cell viability, neuronal excitability, and arborization [31]. Sphingolipids are also engaged in immune phenomena, which critically alter the fate of brain cells in neurodegenerative disorders [31, 37, 38, 42, 43].

**Fig. 2:**
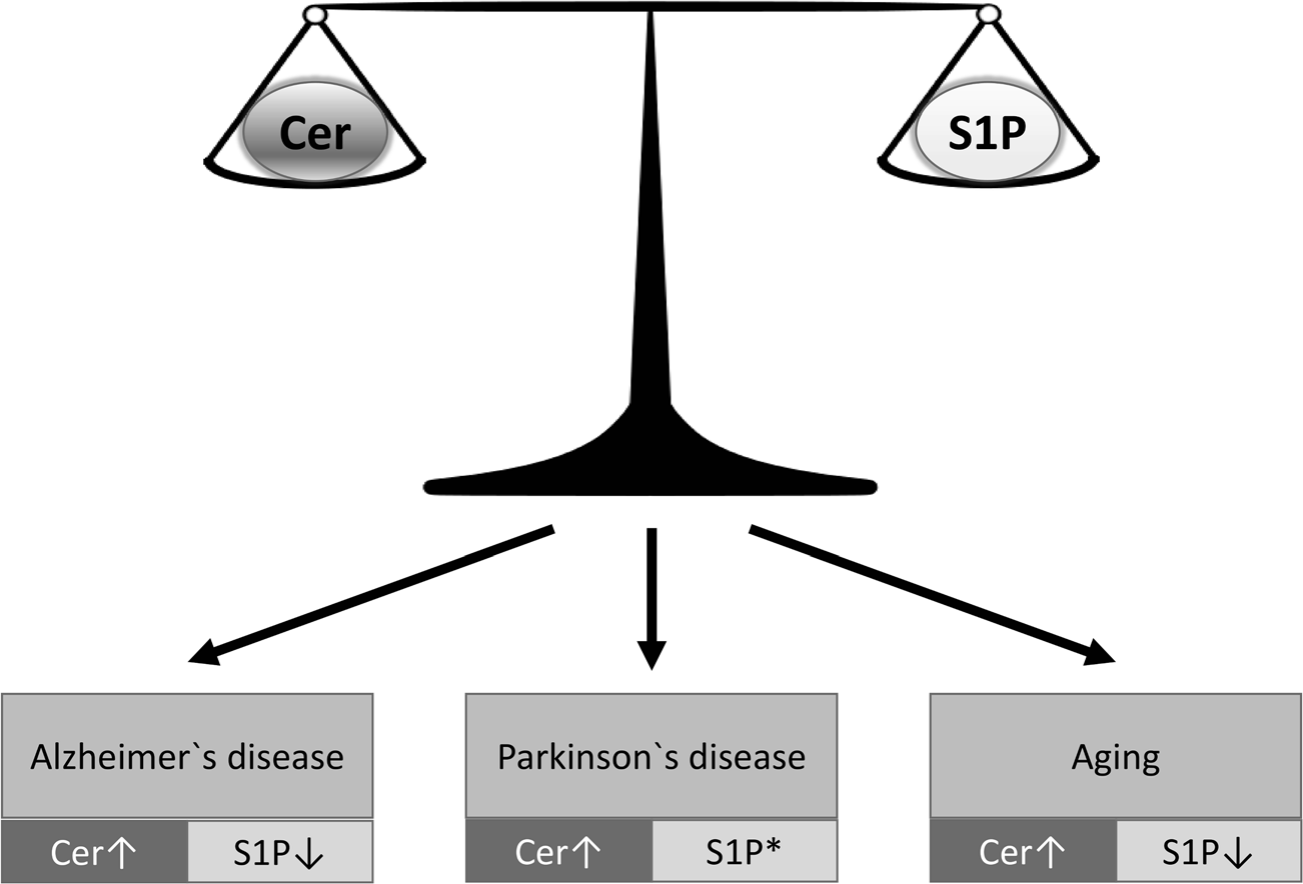
**The changes in bioactive sphingolipid levels observed in aging and neurodegenerative disorders.** Numerous observations in postmortem brain tissues point to the imbalance in the ratio between the concentrations of the apoptosis inducer ceramide, and the typically anti-apoptotic S1P (see Pts. 'Bioactive Sphingolipids in the Pathomechanism of Alzheimer’s Disease' and 'The Role of Bioactive Sphingolipids in Parkinson’s Disease'). Much fewer works address the problem of physiological brain aging, where the changes appear to be partially gender-specific [44].The asterisk indicates clinical data about S1P in PD is missing; however, in experimental disease models, reduced SPHK activity leading to loss of Akt signaling was observed [45, 46].

The roles of S1P and ceramide in the survival of brain neurons are far more complex than the antagonism described in the sphingolipid rheostat model (Fig. 2) and dependent on the signaling *milieu* (see below). However, it is highly probable that the AD-linked changes in the metabolism of bioactive sphingolipids should significantly alter the rates of neurodegeneration.

**Fig. 3:**
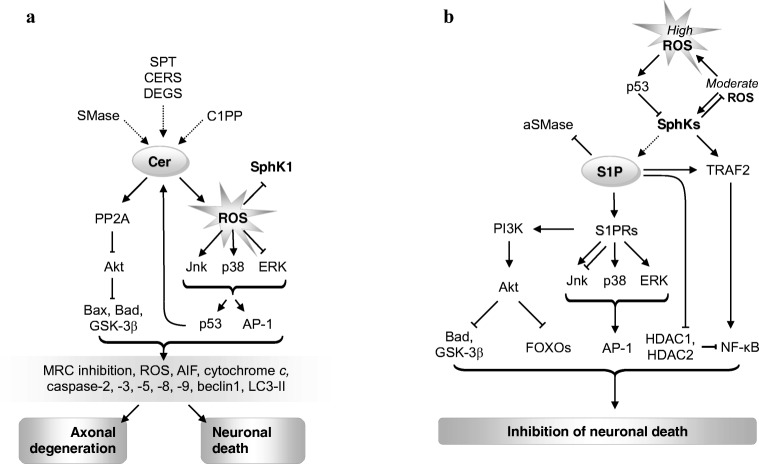
**The role of ceramide and S1P in neurodegeneration pathways.** **a** Ceramide-induced axon loss and neuronal apoptosis. According to [37], modified. **b** The roles of S1P and SPHKs in the modulation of neuronal death. According to various authors (see text). Both S1P and ceramide(s) exert major part of their opposing influence on cell survival through multi-level modulation of the PI3K–Akt pathway, which integrates sphingolipid-based signals with clues on the metabolic condition of the cell, stress levels, etc. [8, 37, 47]. Moreover, sphingolipid signaling displays links with the transcription factors AP-1 and NF-κB [48–52], which regulate a plethora of processes including cell death and inflammation. Akt targets FOXO1a, 3a, 4, and 6 are engaged in cell death regulation in human tissues [53, 54]. The prevailing role of elevated ceramide in cell degeneration/death is mediated by multiple signals: inhibition of mitochondrial respiration and increased production of reactive oxygen species [55]; the release of AIF, cytochrome *c*, or SMAC from mitochondria; the Bcl-2-binding protein beclin1; autophagosomal LC3-II (which binds mitochondrial ceramide to induce lethal mitophagy) [56–58]. While inhibition of HDAC1 and -2 is engaged in the pro-survival signaling of S1P, the role of HDAC3 is more ambiguous [59–62]. AIF, apoptosis-inducing factor; AP-1, activator protein-1; aSMase, acid SMase; LC3-II, lipidated microtubule-associated protein 1 light chain 3β; C1P, ceramide-1-phosphate; C1PP, C1P phosphatase; CERS, ceramide synthase; DEGS, dihydroceramide desaturase; ERK, extracellular signal-regulated kinase; FOXO, forkhead box protein O; GSK-3β, glycogen synthase kinase 3β; HDAC, histone deacetylase; Jnk, c-Jun N-terminal kinase; MRC, mitochondrial respiratory chain; NF-κB, nuclear factor κB; PI3K, phosphoinositide 3-kinase; PP2A, protein phosphatase 2A; ROS, reactive oxygen species; S1P, sphingosine-1-phosphate; S1PR, S1P receptors; SMAC, second mitochondria-derived activator of caspases; SMase, sphingomyelinase; SPHK, sphingosine kinase; SPT, serine palmitoyltransferase; TRAF2, TNF receptor-associated factor 2

In most situations, S1P and ceramide antagonistically signal cell survival or death largely via shared mediators (Fig. 3a, b). Yet more strikingly, both can directly inhibit each other’s synthesis [63, 64]. S1P is classically viewed as an anti-apoptotic agent [65, 66] and has been shown to mediate the actions of numerous anti-apoptotic compounds (Fig. 3b, reviewed in [67]). S1P typically opposes the pro-apoptotic role of ceramide presumably by decreasing oxidative stress and modulating the expressions of pro- and anti-apoptotic proteins of Bcl-2 family (Fig. 3) [47].

S1P can activate p38, ERK, and block Jnk in various tissues by acting through its surface G protein-coupled receptors [67] (however, S1P influence on Jnk can be more varied [68]) (Fig. 3b). ERK appears to mediate the pro-survival action of S1P [69]. S1PRs also stimulate the anti-apoptotic PI3K-Akt pathway [70], whose disruption in AD may heavily contribute to the disease pathomechanism [71, 72]. The nuclear transcription factors targeted by S1P-sensitive pathways include FOXO3a (inhibited by the PI3K-Akt pathway [70]), AP-1 (a transcription factor receiving input from Jnk/p38/ERK [37, 73] and engaged in the network of mutual co-regulation between sphingolipid-related genes [48–50]), or NF-κB (nuclear factor κB, through direct interaction between SPHK1 with TNF receptor-associated factor TRAF2, and through S1P acting as TRAF2 cofactor [74, 75]). Moreover, histone deacetylases (HDAC1 and -2) are inhibited through S1P binding [59] and can block NF-κB via its deacetylation [76]. NF-κB influence on cell death may vary depending on the signaling context, immune activation, etc.

Through PI3K-Akt, S1PRs can inhibit GSK-3β (the crucial tau kinase [77]) and the pro-apoptotic protein Bad. In addition, S1P has been shown to inhibit ceramide production by acid sphingomyelinase (aSMase) [63]. However, contrary to the initial view on the clear-cut S1P-vs.-ceramide opposition, in some situations, S1P may actually exert neurotoxic influence—depending on the spatiotemporal control of its production and degradation, or when its concentration reaches too high levels [78]. Moreover, it is necessary to bear in mind the mentioned ambiguous nature of some of S1P’s mediators: AP-1 [79], ERK [80, 81], or NF-κB [82, 83].

The roles of ceramides in cellular homoeostasis reach far beyond just being pro-apoptotic molecules. Loss of physiological ceramide concentrations can lead to structural disturbances in mitochondria and reduced respiration [84]. Ceramides also regulate membrane dynamics, thus influencing other aspects of organellar function and life cycle such as mitochondrial fusion and fission, or vesicular transport [85]. It is also well understood that their (patho)physiological roles are highly dependent on their chain length/CERS isoforms [86, 87]. When signaling apoptosis, ceramides appear to use a spectrum of mediators largely shared with S1P, albeit often in a contrasting way (Fig. 3a). Ceramides lead to dephosphorylation and inactivation of Akt via the protein phosphatase PP2A; this relieves Akt’s inhibitory influence on Bad and GSK-3β [77]. On the other hand, ceramide-associated increase in reactive oxygen species (ROS) leads to the activation of p38 and Jnk and to ERK inhibition [37]. The combined influence of p38, Jnk, and ERK modifies the activities of p53 and AP-1 (c-Fos, c-Jun) transcription factors [88]. Together with Bad and GSK-3β activation, these changes cause mitochondrial alterations and via cytochrome *c* release and the activities of caspase-2, -3, -5, -8, and -9 may lead to axonal degeneration or neuron death [37]. Ceramides might also directly form pores in the outer mitochondrial membrane, leading to the release of cytochrome c and other proteins [89]. Other mitochondrial mediators of apoptosis known to be released in neurons by ceramides include apoptosis-inducing factor (AIF), the second mitochondrion-derived activator of caspase (Smac), and the stress-regulated endoprotease Omi [56]. Ceramide-induced apoptosis thus involves both caspase-mediated and caspase-independent pathways.

As discussed above, changes in the balance between S1P and ceramide (Figs. 2 and 3) may not only influence apoptosis but also change the regulation of autophagy by the complex interplay between mTOR, beclin, and Bcl-2. S1P-dependent autophagy is thought to be a homeostatic, pro-survival response involved in the clearance of intracellular debris (damaged proteins/dysfunctional organelles) [67]. In AD, autophagy can play critical role in the defense against oxidatively damaged cellular components, and its disturbances may exacerbate Aβ and tau deposition [90, 91]. However, autophagy can also constitute a mode of cell death, where autophagolysosomal degradation of mitochondria is dependent on the interaction between ceramide and LC3-II (lipidated microtubule-associated protein 1 light chain 3β) present on lysosomes [57] (Fig. 3).

### Bioactive Sphingolipids in Aging

Bioactive sphingolipids have been investigated in the course of aging and in association with extreme longevity [27, 92, 93]. Centenarians display altered fatty acid pattern in ceramides and glucosylceramides—higher levels of sphingolipid species possibly linked to stress resistance (low oxidation susceptibility due to unsaturated fatty acid content) [27]*,* while increased concentration of sphingomyelins (ceramide precursors) has been observed during aging [94]. Sphingolipids appear to have significant influence on the course of aging; research on lower organism models suggests links between ceramide synthesis and longevity [8, 25, 95–97]. Importantly, bioactive sphingolipids are capable of influencing the IIS (insulin/insulin-like signaling)–PI3K–Akt, a highly conserved, versatile modulator of metabolism, aging, and stress response (Fig. 3) [98–102]*.* IGF-I signaling in the brain has been identified to negatively influence organism longevity also in mammals [103, 104], although some controversies persist [105]*.* Results obtained in humans appear to support IIS role in aging [106, 107]*.* IIS seems to redirect the vital resources away from long-term investment in favor of more current needs such as metabolic regulation and cellular survival. This leads somewhat surprisingly to the trophic influence of IIS in the brain [8, 108–111]*.* PI3K-Akt signaling regulates SPHKs and S1PRs expression/activity and intracellular sphingolipid transport [112–115]. In turn, S1P receptors can differentially modulate Akt activity [116–118]. Ceramide leads to inhibition of Akt-dependent pro-survival signaling [47, 119, 120], while C1P stimulates it [121, 122]*.*

The links between sphingolipids and cellular stress are an extremely important aspect of their potential involvement in aging (Fig. 3) [8, 67]. SPHK1 might inhibit ROS and reduce sensitivity to DNA damage [123]. S1P and ceramides are under positive influence of the stress sensor p53, and faulty ROS control leads to alterations in S1P/ceramide signaling [67, 124–126]*.* Even more than in aging, stress and inappropriate stress responses are central elements of the pathomechanism of neurodegenerative disorders.

## Bioactive Sphingolipids in the Pathomechanism of Alzheimer’s Disease

The pathogenesis of AD is not yet fully elucidated, and the actual roles of many of the observed disturbances are not clear. Accumulating evidence points to the involvement of bioactive sphingolipids in AD starting from the earliest, prodromal stages [127].

Well-documented mechanisms that induce neuronal and synaptic degeneration in AD brain include the following: oxidative damage, altered redox signaling, mitochondrial dysfunction, glucose hypometabolism/other metabolic stresses, Ca^2+^ deregulation, and inflammatory response. Many of these pathways are triggered and propagated due to the actions of soluble oligomers of Aβ peptide on neurons and glia. The role of ceramide/S1P was analyzed in the context of these damage pathways as well as the process of amyloidogenesis.

### The Interactions Between Ceramide/S1P and AβPP/Aβ Metabolism

Structural roles of sphingolipids in cellular membranes including lipid rafts constitute an important aspect of their engagement in AβPP/Aβ metabolism [128]. Lipid rafts are cholesterol- and sphingolipid-enriched microdomains of the plasma membrane described as *signaling platforms* [129, 130]*.* Rafts are strongly associated with Aβ production, and both *β-* and *γ-*secretases are enriched in these structures [129, 131, 132]*.* Lipid rafts also seem to influence Aβ aggregation [133]. In turn oligomeric Aβ_42_ associates with rafts [134]; Aβ can change membrane fluidity, which may exert a feedback influence on its own production [135]*.*

Rafts are sensitive to fluctuations in sphingolipid levels, leading, e.g., to changed properties of membrane-associated enzymes or receptors. Sphingolipid/ceramide deficiency leads to increased secretion of sAβPPα, the product of non-amyloidogenic cleavage. However, it also leads to enhanced secretion of Aβ_42_ possibly through modulation of raft-associated proteins and changes in raft membrane properties resulting in altered *α*- vs. *β*-cleavage ratio [136]. Exogenous addition of ceramide and elevated endogenous ceramide increased the level of Aβ. C6-ceramide, a cell-permeable ceramide analogue, increased the rate of Aβ biosynthesis by affecting *β*-cleavage of AβPP. Lipid raft ceramides stabilize BACE1 (β-site AβPP cleaving enzyme 1, a β-secretase) [137]. Additionally, it was shown that synthetic ceramide analogues may also function as γ-secretase modulators that increase Aβ_42_ production [138]. FTY720 in turn has been demonstrated to reduce hippocampal neuron damage and the resulting learning and memory deficits in a rat model induced by bilateral, stereotactic injection of pre-aggregated Aβ_42_ into the hippocampus [139]. Some of the neuroprotective effect might be ascribed to mobilization of extrasynaptic, N-methyl-D-aspartate receptors to the synapse (a phenomenon reducing cellular sensitivity to Aβ-induced neurotoxic calcium influx) [140]. Importantly, FTY720 and KRP203 (another SPHK2 substrate that can bind S1PR upon phosphorylation) have been shown to reduce neuronal Aβ generation [141]. However, the relationship between S1P and AβPP metabolism is still obscure, as the compounds used may as well downregulate S1PR-dependent signaling; moreover, FTY720 increased Aβ_42_ in mice in addition to reduction in Aβ_40_ [141]. Positive correlation between S1P production by SPHK2 and AβPP processing has been reported [142]. S1P produced by SPHK2 may activate BACE1, thus leading to higher release of Aβ peptides. S1P was shown to specifically bind to full-length BACE1 and to increase its proteolytic activity. The production of Aβ peptides can be reduced in N2a neuroblastoma cells by pharmacological inhibition of sphingosine kinases, homozygous *SPHK2* gene deletion, or overexpression of the S1P lyase gene and *SGPP1* phosphatase [142]. A shift in SPHK2 subcellular distribution from cytosol to the nucleus was observed to correlate with Aβ deposition in AD brains by Dominguez and collaborators [143]. In turn, Aβ production correlates with low SPHK1 and high S1P lyase protein [144]. Disturbances in S1P observed in AD may not only critically regulate caspase-mediated AβPP cleavage. S1P regulates lysosomal AβPP metabolism in a calcium-dependent manner [145]. S1P is also a pro-secretory molecule, and the dependence of AβPP secretion on S1P signaling has direct potential significance in AD [146]. The regulation of gene expression via S1PRs and through intracellular signaling can also lead to complex changes in cellular metabolism. AβPP modulates this process, as shown in FTY720-treated mice overexpressing mutant (V717I) AβPP. FTY720, which raises significant hopes as a repurposed neuroprotective drug in AD, increased the gene expression of sphingosine kinases (*SPHK*s), ceramide kinase (*CERK*), and the anti-apoptotic Bcl-2 in an age-dependent manner [147].

**Fig. 4:**
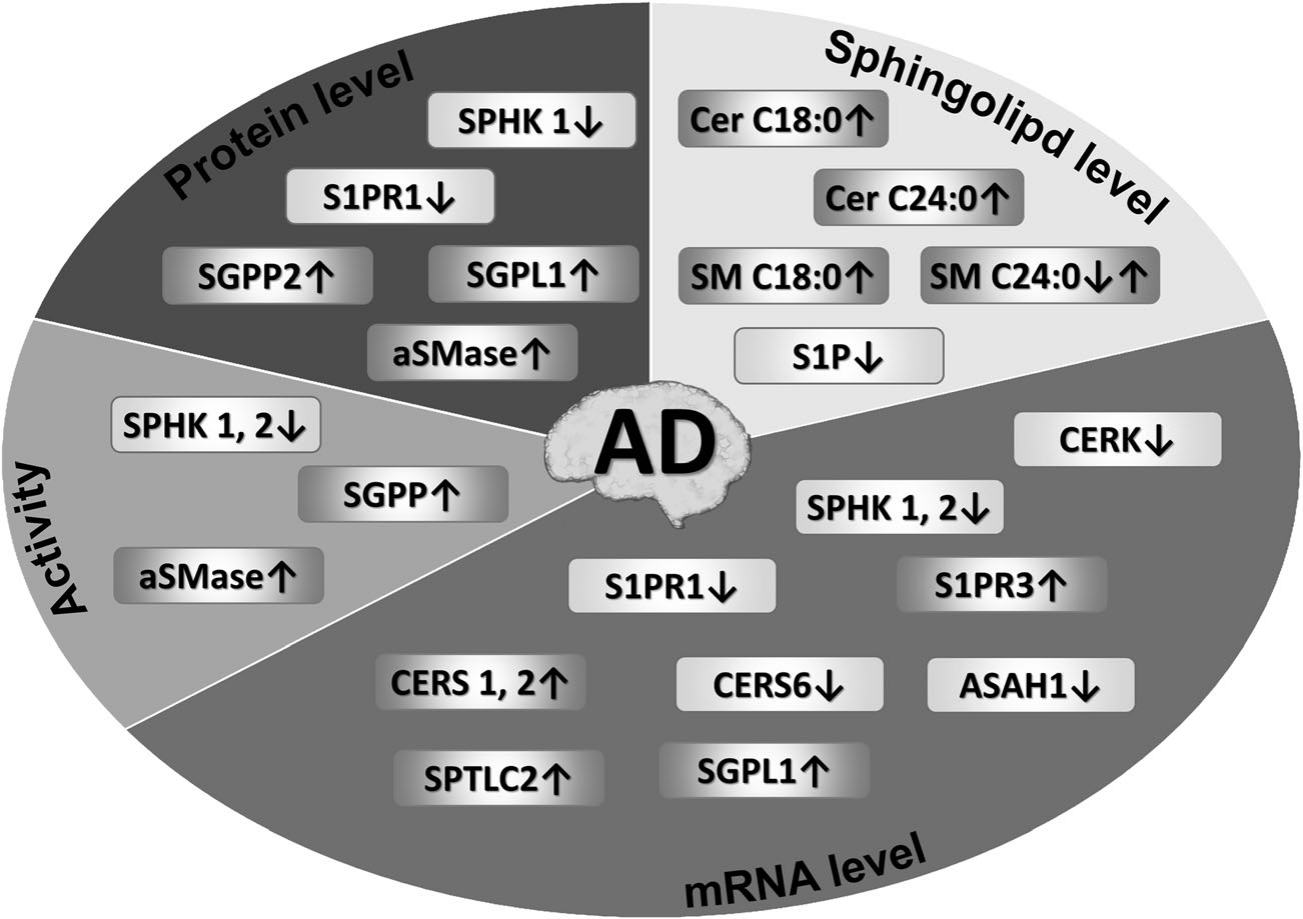
**Changes in brain sphingolipid metabolism and signaling observed in Alzheimer’s disease.** Human postmortem brain material was used by numerous authors to compare the levels of bioactive sphingolipids, mRNAs, proteins, and enzyme activities. In the hippocampus S1P levels, mRNAs for *CERK*, *S1PR1*, *SPHK1*, *SPHK2*, and SPHK activity are reduced [65, 147, 148]. Lower S1P levels, S1PR1 protein, and SPHK protein/activities were observed along elevated S1P lyase proteins in selected cortical areas and in the hippocampus [65, 144]. Increased ceramide (Cer) and sphingomyelin (SM) levels, mRNAs for ceramide synthases (*CERS1*, *2*), SGPL1, SPTLC2, aSMase, and sphingomyelinase protein and activity were observed in brain cortical areas, while *ASAH1*, *CERK*, and *CERS6* mRNAs were reduced [148–151]

The *milieu* of the brain affected by AD provides multiple stressors such as ROS and cytokines, which could in turn lead to increased ceramide production (Figs. 3 and 4). Lee et al. [152] showed ceramide-dependent death of oligodendrocytes induced by Aβ. Activation of neutral sphingomyelinase (nSMase) and increase in the level of ceramides was observed. Moreover, it was also shown that suppression of ceramidase activity additionally increased the toxicity of Aβ [152]. In the studies of Jana and Pahan [153] superoxide-mediated activation of nSMase was observed in primary culture of human neurons treated with Aβ_1–42_. NAPDH oxidase mediated the effect, because gene silencing for the p22phox subunit by antisense oligonucleotides inhibited the apoptosis of neurons induced by Aβ_1–42_. The authors showed that the use of N-acetylcysteine and the NADPH oxidase inhibitor prevented the generation of ceramides and protected against neuron apoptosis. Similar results were obtained in primary rat cortical neurons treated with Aβ oligomers where an increase in neutral and acid sphingomyelinase activities was observed [63]. In another study, Gomez-Brouchet et al. [154] indicated that exposure to Aβ_25–35_ induced strong SPHK1 inhibition and ceramide accumulation in neuronal SH-SY5Y cells. In that study, the cell death was prevented by overexpression of sphingosine kinase, whereas downregulation of the enzyme by RNA interference enhanced the cell death. Inhibitors of both SMases and exogenously administered S1P demonstrated cytoprotection in the model. Ayasolla et al. [155] observed that in the primary culture of rat astrocytes treated with TNF-α/IL-1β, followed by Aβ_25–35_, there is a greater increase in the expression of induced nitric oxide synthase (iNOS) and nitric oxide production (NO) than in TNF-α/IL-1β-only astrocytes. In a glial cell line treated with Aβ_25–35_ and LPS/IFNγ, an increase in iNOS expression and NO production was observed. These phenomena were accompanied by an increase in the level of ceramides as a result of the activation of nSMase. Similar results were obtained in oligodendrocytes treated with Aβ_25–35_ and TNF-α, or with C2-ceramide and TNF-α [156]. TNF-α-induced ceramide production was also observed by Martinez et al. [157].

The accumulated oligomerized Aβ peptide in AD brain may also promote ceramide formation, as demonstrated both in cell culture [148, 153–156] and animal models [158]. Imbalance in mRNA expression of enzymes responsible for S1P to ceramide ratio, which potentially might decide of the brain cell fates, is observed from the earliest clinically recognizable AD stages (Fig. 4) [149]. Sphingomyelin hydrolysis stimulated by Aβ appears to be the main source of ceramides in the pathology of Alzheimer’s disease [155, 159, 160], along with de novo synthesis (expression of the de novo enzymes increases gradually during AD progression [149]). Activation of SPT by Aβ peptides has been observed, resulting in neurotoxic increase of ceramide levels via de novo pathway [148, 161]. Aβ induces apoptosis by activating aSMase and nSMase, thereby contributing to the increase in ceramide levels. Senile plaques contain aSMase and nSMase proteins along with high levels of saturated ceramides [63, 162], and aSMase activity is upregulated in human AD brains (Fig. 4) [150]. Examples of genes upregulated by AD also included ceramide synthases *CERS1* and *CERS2*, S1P lyase *SGPL1*, or serine palmitoyltransferase catalytic subunit *SPTLC2*, while the acid ceramidase *ASAH1*, ceramide kinase *CERK*, or—less obviously—*CERS6* were reduced [131, 149]. However, contrary to the abovementioned results, Couttas et al. [28] have found an early loss of CERS2 activity at Braak stage I/II (temporal cortex) to III/IV (frontal cortex, hippocampus). An interesting but underexplored link has been identified between the still obscure physiological AβPP role and sphingolipid metabolism, as AβPP intracellular domain is capable of reducing the expression of *SPTLC2*, potentially keeping the whole sphingolipid metabolism under negative control [131]. Aβ also disturbs S1P signaling, potentially shifting the balance towards a much more pro-apoptotic state (Figs. 2 and 4) [148]. Aβ can downregulate the genes for SPHKs and diminish the level of S1P as observed in wild-type and AβPP-overexpressing PC12 cells in culture [163]. The study by Couttas et al. [65] showed reduced S1P levels with increasing Braak stage in tissue samples taken from the CA1 region of the hippocampus, or gray and white matter of the inferior temporal gyrus (Fig. 4). AD brains also display upregulated expression of S1P lyase SGPL1 and S1P-metabolizing phosphatases [65, 149]. The studies of Ceccom et al. [144] showed a decrease in immunoreactivity of SPHK1, S1P receptor 1, and an increase in S1P lyase in samples taken from the frontal and entorhinal cortices from human AD brains. However, the complex influence of SPHK2 signaling on cell fate and neurodegeneration is reflected by the study of Takasugi et al. [142] who reported upregulation of SPHK2 activity in AD brain cortex while other authors reported reduction of its activity and mRNA in the hippocampus [65, 147].

Importantly, SPHKs’ roles include engagement in the regulation of inflammation, a phenomenon already exploited in the therapy of relapsing remitting multiple sclerosis [164]. The known engagement of sphingolipids in the modulation of NF-κB signaling by TNF-α [165] and other factors strongly suggests widespread opportunities in this field. Aβ specifically modulates the expression of some S1P cell surface receptors in monocytes [166]. Sphingolipid modulators inhibit the accumulation of mononuclear phagocytes in response to Aβ, leading to proposals of their use as therapeutic agents [166]. In turn, anti-ceramide immunity might also contribute to the disease progression [167].

An important hint about the significance of sphingolipids in AD is the association of apolipoprotein E (ApoE whose polymorphisms are strongly linked to AD risk [168]), with the receptor-mediated signaling of secreted S1P [169]. Moreover, correlation has been observed between SPHK activities/S1P content and ApoE allele (2.5× higher S1P/sphingosine ratio in the hippocampus of ApoE2 vs. ApoE4 carriers) in AD [65]. Sphingolipids might be useful, accessible AD biomarkers [170–172] and—potentially—therapeutic targets [173].

### S1P/Ceramide and the Exosome-Mediated Spread of AD Pathology

Exosomes are sphingomyelin- and ceramide-enriched vesicles created inside the multivesicular endosomes (MVE) and then secreted when MVE membrane fuses with the plasmalemma. Exosomes are engaged in intercellular communication and carry microRNAs (miRNAs), messenger RNAs (mRNAs), and protein- and lipid-based signaling molecules. Vesicles released by Aβ-treated astrocytes contain the pro-apoptotic prostate apoptosis response 4 (PAR-4) protein and cause apoptosis in naive cultures [174]. Rodent exosomes can contain Aβ, BACE1, and presenilins 1 and 2 [175]. Amyloid plaques in the AD brain contain an exosome marker [176]. These results have led to a hypothesis that exosomes might seed Aβ aggregation [177]. However, at least under some circumstances exosomes can also inhibit Aβ oligomerization and promote its microglia-mediated clearance [178]. These results might explain the observed association of exosomes with Aβ as a physiological, neuroprotective phenomenon [179], at least in the healthy tissue. It is also possible that exosomes of various origin (e.g., neuronal vs. astrocyte) might exert opposite influence or that the exosomal membranes might facilitate Aβ aggregation independently of protein-mediated exosomal functions (e.g., Aβ degradation by exosomal insulin-degrading enzyme or neprilysin)—reviewed in [177]. Additionally, exosomes can serve as a vehicle for the extracellular secretion and cell-to-cell transport of ASN and tau protein, potentially further supporting the spread of aggregation pathology [180, 181]. S1P receptor signaling has been implicated in exosomal cargo sorting: activity of the S1PR-regulated Rho family GTPases was necessary for the process, and Gβγ inhibitor blocked it [182]. Exosome secretion can be modulated by the activity of neutral sphingomyelinase 2 (nSMase2) and sphingomyelin synthase 2 (SMS2), suggesting unique roles for these enzymes in AD [178, 183], and additional significance for the disturbed ceramide levels observed in the course of the disease, as discussed above.

## The Role of Bioactive Sphingolipids in Parkinson’s Disease

The selective, spatially progressing neurodegeneration observed in PD defies full explanation, although hypotheses have been created that probably successfully identify and describe important aspects of its mechanism [4]. Pathological aggregation of ASN inside neuronal cells is widely associated with PD; ASN might also play some role in AD [184]. ASN binds lipid rafts, and negatively regulates S1PR1 signaling there [130]. Moreover, the relatively recently recognized phenomenon of ASN secretion suggests links with sphingolipid signaling, as the engagement of sphingolipids in neuronal secretory pathways is well documented [7, 146]. ASN may undergo regulated secretion in a number of partially characterized mechanisms [45, 185–190], possibly leading to the peptide being functionally “addressed” for different destinations, allowing passage of ASN (also oligomeric) into various compartments of recipient cells [191]. This may have high significance for the postulated spread of ASN-induced pathology along anatomical connections [4].

**Fig. 5:**
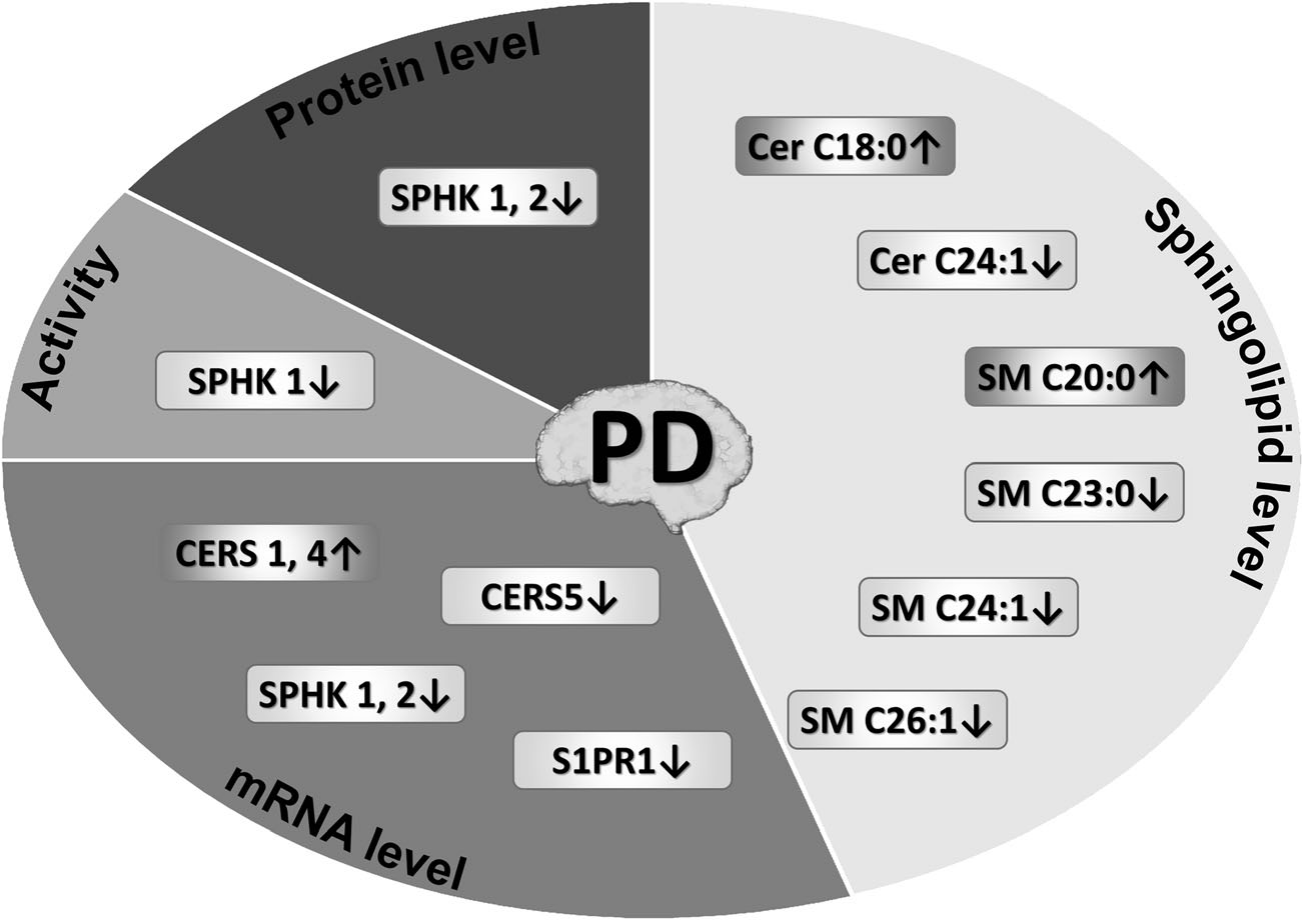
**Changes in brain sphingolipid metabolism/signaling observed in Parkinson’s disease and its experimental models.** Ceramide synthase (CERS) mRNAs, ceramide (Cer), and sphingomyelin (SM) species were measured in postmortem PD brains by Abbott et al. Increased *CERS1* expression was found along with a shift towards shorter ceramide acyl chain lengths in brain regions most affected by the disease, although reduction in total levels of ceramide and sphingomyelin concentration was observed [192]. Reduced sphingosine kinase-1 and -2 (SPHKs) and S1P receptor 1 (*S1PR1*) expression was observed in the mouse MPTP (1-methyl-4-phenyl-1,2,3,6-tetrahydropyridine)-induced model [46, 193]. The MPTP model also displayed lower protein levels and activity of SPHK1 [193, 194]

Recent evidence suggests links between sphingolipids and PD although data is relatively less abundant. PD is associated with disturbances in sphingolipid metabolism (Figs. 2 and 5) [192, 195, 196, reviewed in 12]. Lipidomic analysis has shown that the levels of ceramides and sphingomyelins were altered in postmortem PD brain tissue as compared to the control samples (a tendency towards shorter acyl chain) [192]. Findings in body fluids suggest the diagnostic value of sphingolipids in PD [197]. In blood plasma, several saturated ceramides and one unsaturated species were significantly higher in PD [198]. Additionally, some blood ceramide species were higher in PD with dementia than in non-demented PD patients and the levels of several saturated ceramides associated with PD-linked psychiatric complications [198, 199]. Mutations in the *SMPD1* gene have been repeatedly confirmed to correlate with PD risk [200–204]. The expression and activity of SPHK1 are reduced in MPTP (1-methyl-4-phenyl-1,2,3,6-tetrahydropyridine)-induced murine model of PD (Fig. 5) [194], which can lead to enhanced ROS as well as BAX and HRK (harakiri) mRNA expression [205]. Importantly, the possible similarities between AD- and PD-linked disturbances of sphingolipid metabolism are not limited to direct regulation of apoptotic signaling; products of S1P degradation have been found to modulate autophagic/lysosomal degradation of both Aβ/AβPP and ASN [206]. Administration of FTY720, a sphingosine analog metabolized in the tissue into a S1P receptor modulator, protected against neurodegeneration and behavioral defects in mouse PD models induced by MPTP, 6-hydroxydopamine, or rotenone—via S1PR1 and probably Akt [194, 207, 208]. In turn, secreted ASN can inhibit S1PR1 signaling and disturb the receptor’s localization in lipid rafts [130]. It is worth mentioning, however, that FTY720 failed to offer protection in a model of PD induced by subacute (5 days) MPTP administration [209].

The PD-linked changes may exert influence not only on neuronal survival and phenotype, but also potentially on the central mechanisms of PD pathology. Sphingomyelin has been demonstrated to modify the expression levels of ASN [210]. Degradation of overexpressed or otherwise pathologically altered ASN may be dependent on the sphingomyelinase [211]. Pharmacological inhibition of SPHK1/-2 activities in cells treated with low concentrations of MPP^+^ leads to enhanced secretion of ASN, which may strengthen the significance of the new, still underestimated mechanism of Parkinsonian pathology [45]. Outside the CNS, FTY720 also reduced ASN burden in the enteric nervous system, improving gut motility whose reduction is an early peripheral symptom in PD [212]. Interestingly, pramipexole (a dopamine D2/D3 receptor agonist) reversed SPHK1 inhibition in the MPTP model [194], suggesting further interactions between sphingolipid and dopamine signaling.

Glucocerebrosidase (GBA) is a lysosomal enzyme that produces ceramide from glucocerebroside (glucosylceramide) [196]. GBA deficiency/mutations are among top genetic contributors to the development of PD [196, 213, 214] and are statistically associated with Parkinson’s disease [215, 216], contributing to its early development, rapid progression, and presence of additional psychiatric symptoms [217, 218]. Interestingly, β-glucocerebrosidase activity is reduced in the cerebrospinal fluid (CSF) of PD patients even if they do not carry any *GBA1* mutations [219]. Variants in the *GBA* gene may be highly useful in the prediction of PD course [220]. Accumulation of glucosyl compounds and cholesterol has attracted most attention as the pathomechanism in GBA mutations/deficiency [221], but changes in ceramide levels cannot be excluded as an important contributing factor [214, 222]. The enzyme is important in ASN degradation [223] and appears to protect against ASN aggregation [224]. Additionally, PD patients not carrying the GBA mutation also display elevated glucosylceramides in their plasma [198]. Small-molecule GBA chaperones have been suggested as a possible means of therapy in synucleinopathies [225].

## Sphingolipids in Huntington’s Disease and Amyotrophic Lateral Sclerosis

In recent years, data has been accumulating on the engagement of sphingolipids in Huntington’s disease (HD) and amyotrophic lateral sclerosis (ALS). HD is a neurodegenerative brain disorder involving striatum and cortex and manifesting itself in motor and cognitive disturbances. It is caused by a dominant mutation, a triplet expansion in the huntingtin (HTT) gene. HD appears to disturb sphingolipid metabolism; increased SGPL1 protein has been observed in the cortex and striatum of advanced HD postmortem brains, accompanied by striatal reduction in SPHK1 [226]. Most data, however, has been obtained from animal models. Results suggest, similarly to other neurodegenerative disorders, that imbalance in sphingolipid concentrations and enzyme expression levels occurs in early stages of the disease development [227–230]. SPTLC1 and CERS1 were reduced in the brains of R6/2 mice [227], a HD model mice transgenic for the first exon of huntingtin harboring ca. 160 CAG repeats [231]. The altered sphingolipid metabolism has also been noted in a variety of other cellular and animal HD models [226], although the changes seem to be less clearly weighted towards cell death than in, e.g., AD [228]. However, the reduced S1P levels observed in R6/2 mice [226] appear to be a relevant potential therapeutic target, as FTY720 has been demonstrated to improve neuronal activity, reduce brain atrophy, improve motor function, and increase R6/2 animal survival [232]. Moreover, S1PR agonists increased huntingtin phosphorylation and reduced its aggregation [232, 233]. FTY720 also increased the levels of brain-derived neurotrophic factor (BDNF) levels and mitigated the upregulation of NF-κB, iNOS, and TNF-α that would otherwise lead to the potentially neurotoxic activation of astrocytes; FTY720 thus preserved synaptic plasticity and memory in R6/1 mice (another model with lower number of glutamine repeats in the first huntingtin exon) [234]. The S1PR5 stimulator A-971432 preserved blood-brain barrier integrity in R6/2 mice [233]. These results have led to proposal of S1P-modulating therapy of HD [235].

ALS is a neurodegenerative disorder encompassing motor neuron degeneration, muscle wasting, and paralysis, characterized by severe deregulation of metabolism, including lipid metabolism [236]. Ceramides and their glucosyl and lactosyl derivatives are increased in ALS patient spinal cords [237]. SOD mutant mouse model of ALS displays disturbances in the expression of genes related to immune regulation, exosomal secretion, or lysosomes. Importantly, disturbed levels of ceramides and sphingosine were noted to correlate with disease severity along with the expression of SPHK1, or SGPP2 and sphingolipids—sphingosine and ceramides (d18:1/26:0) [238]. Increased glucosyl ceramide synthase (GCS) expression might hamper normalization of oxidative metabolism and motor recovery [236]. Also in this case, FTY720 improved neurological scores and survival in SOD mutant mice [239]. It modified the mRNA expression of, e.g., iNOS (reduced by the treatment), ARG1 (increased), BDNF (increased), and interleukin genes (IL-1β reduced, IL-10 increased), despite administration starting in the symptomatic phase [239].

## MicroRNA Signaling and Bioactive Sphingolipids in Neurodegenerative Disorders

miRNAs are increasingly viewed as central regulators of neuronal homoeostasis, and their causal roles in neurodegenerative disorders are rapidly gaining attention. Deregulation of miRNA-based gene expression control may be a novel disease mechanism, but also delivers potentially valuable biomarkers of its development [240–242]. Considerable research interest has been generated concerning the role of miRNAs in the neuropathology of bioactive sphingolipids in several progressive age-related human neuropathological diseases, and especially how specific miRNAs may contribute to the dynamic molecular-genetic processes involving aberrant ceramide/C1P/S1P metabolism in both AD and PD. In humans, miRNAs are a family of 18–22 nt single-stranded RNAs that posttranslationally interact with, and regulate, the expression of mature mRNAs. Single upregulated miRNA can target multiple mRNAs to reduce their expression, and multiple miRNAs can target a single mRNA [243–245]. Whenever progressive neurodegeneration is encountered in central nervous tissues undergoing pathological change, progressive neuronal atrophy and brain cell death the NF-κB-sensitive, pro-inflammatory and potentially pathogenic miRNA species such as miRNA-34a, miRNA-146a, miRNA-155, and several others have been shown to be abundant (and readily detectable by hybridization methodologies) in the cytoplasm of degenerating neurons, as well as in both the extracellular fluid (ECF) and CSF, which is contiguous with ECF. While these miRNAs are normally required for the homeostatic operation of brain cellular and membrane-signaling functions, their upregulation and persistence in deteriorating nervous tissues and the nature of their interaction with biological membranes is associated with, and indicative of, the propagation and spreading of neurodegenerative disease. These miRNAs may be a diagnostic tool for the cytoplasmic status of brain cells at risk for neurodegeneration [241–243].

In turn, the upregulated microRNAs such as miRNA-34a, miRNA-146a, and miRNA-155 appear to antagonize both individual mRNAs and small families of functionally related mRNAs and, in doing so, affect entire systems of CNS-membrane-relevant genes and plasma membrane processes. Indeed, overexpression of miRNA-34a, miRNA-146a, and/or miRNA-155 have been shown to affect the expression of a large number of genes normally involved in glucose metabolism, innate-immune regulation, membrane integrity, normal vascular function and endothelial cell permeability, oxidative phosphorylation, synaptic plasticity, and exosome generation, encapsulation, and release. All of these processes have been shown to be altered in AD- and PD-affected brain cells and tissues [177, 244–250]. Examples of target mRNAs include [244, 245, 248]:

- Immune regulators such as NF-κB, interleukins 4 and 17a, interleukin-1 receptor-associated kinase-1 (IRAK-1), or complement factor-H (CFH)
- Neuronal activity/synaptic plasticity/scaffold genes such as glutamate receptor genes NR2A, GluR1, synaptobrevin 2, and synaptotagmin 1
- Genes coding for glycolysis and oxidative phosphorylation proteins such as succinate dehydrogenase complex C, ubiquinol-cytochrome c reductase binding protein and ubiquinol-cytochrome c reductase complex III subunit VII (UQCRB and UQCRQ, respectively), or phosphofructokinase-1
- Amyloidogenesis-linked genes such as the membrane protein tetraspanin 12 (TSPAN12), or the master postsynaptic membrane-organizing ankyrin-cytoskeletal protein SHANK3

Put another way, specific pathology-linked miRNAs appear to regulate a large number of plasma membrane-resident and plasma membrane-organizing components whose character is defined by sphingolipid composition, turnover, and metabolism.

Many CNS-abundant miRNAs have, in addition, important regulatory functions in the expression of enzymes involved in the generation of ceramide, sphingosine, C1P, S1P, and/or their receptors, both in healthy brain aging and in neurological disease. For example, the pro-inflammatory and rapidly induced NF-κB-regulated miRNA-155 (encoded in humans at chr 21q21.3) has been shown to regulate biosynthesis of the S1PR1 which functions in the amelioration of pathogenic inflammation in systemic autoimmune disease [246–248, 251]. Interestingly, a five-member cluster of miRNAs encoded on human chromosome 21 that includes let-7c, miRNA-99a, miRNA-125b, miRNA-155, and miRNA-802 may help explain the complex phenotypic diversity of trisomy 21 (Down’s syndrome; DS) and the strong linkage between DS and the aberrant sphingolipid and ceramide metabolism associated with trisomy 21 (DS) neuropathology [252–254]. Interestingly, some of the most recent brain biolipid research describes the association between neurotoxins secreted by the human gastrointestinal (GI) tract microbiome and inflammatory neurodegeneration of nervous tissues, a complex pathogenic process that is certain to involve CNS sphingolipid composition, their organization, and interactive metabolism [255, 256].

As mentioned earlier, sphingolipids are key regulators of exosomal secretion. Their roles include exosome formation, encapsulation, and miRNA shuttling across the plasma membrane [257]. Exosomes and other extracellular vesicles secreted into the extracellular space from both neuronal and glial cells are enriched with the sphingolipid ceramide, as well as other more complex glycosphingolipids such as gangliosides, and may also be enriched in various species of pathogenic or “communicating” miRNAs [177, 243–245]. Such exosomal vesicle-bound miRNAs: (a) should be reflective of the sphingolipid and miRNA composition of the brain cell cytoplasm from which they were originally derived; (b) may serve the role as a novel form of intercellular communication among brain cells; (c) may carry selective miRNA ‘cargos’ that regulate both bioactive sphingosine/S1P and ceramide/C1P metabolism as well as other miRNA-mRNA targets in adjacent cells; (d) have been implicated in the inter-neuronal “spreading” of pathogenic signals via “paracrine” and related secretory effects in the diseased and neuro-degenerating brain; (e) have considerable potential for being clinically useful as a predictor and non-invasive diagnostic marker for AD and/or PD; and (f) may provide a “molecular-genetic” signature for a defined group of miRNAs associated with a particular neurological disease [177, 243–248].

Recently, data on the engagement of miRNA-based gene regulation in HD and ALS begun to accumulate. *Postmortem* HD cortex samples from Brodmann’s area 4 reveal disturbed miRNA expression (reduced miR-9, miR-9*, miR-29b, miR-124a, miR-132) that might stem from loss of huntingtin-transcription factor interaction in neuronal cells [258]. Numerous deregulated circulating miRNAs have been found in HD cases and might reflect not only the ongoing neurodegeneration but also altered communication with the periphery [259]. Links between altered miRNAs and perturbations in apoptotic and cell cycle signaling have been proposed as a possible mechanism of cell loss in HD [260]. The R6/2 mouse HD model displays reduction in miRNA-34a-5p, a member of miRNA-34 family that is engaged in p53- and SIRT1-dependent modulation of cell cycle, senescence, and apoptosis [261]. A series of mouse models with various numbers of CAG repeats in the huntingtin gene has shown a repeat number- and brain part-dependent alteration in miRNA transcriptome (including miRNAs engaged in neuronal development/survival [262]). Altered miRNA levels in blood plasma and CSF have been proposed as HD biomarkers [263, 264].

The engagement of microRNAs in the pathology not only of neurons but also muscles is relatively better characterized in ALS [265]. A high-throughput next-generation sequencing project has identified reduction in the blood levels of 38 miRNAs in sporadic ALS patients, including let-7 and miR-26 families. The pattern of reductions was dependent on the disease phenotypic expression and progression rate, making them potentially useful for diagnostic purposes [266]. In turn, upregulation of miR-223-3p, miR-326, and miR-338-3p observed in tissue bank neuromuscular junction samples of ALS patients may disturb HIF-1 and brain-derived neurotrophic factor signaling [267]. Disruption of the intraneuronal localization of RNAi *machinery* (leading to deregulated axonal protein synthesis) has also been noted as an important aspect of ALS pathology [268]. Moreover, degenerating/dying neurons release miRNA-218 which leads to changes in astrocyte phenotype such as reduced expression of excitatory amino acid transporter 2 or peroxisome proliferator-activated receptor gamma coactivator 1α and to astrogliosis which likely contributes to the neuron loss [269].

## Concluding Remarks

Neurodegenerative disorders belong to the most widespread, devastating, and uncontrollable diseases. Alzheimer’s, Parkinson’s, and Huntington’s diseases and amyotrophic lateral sclerosis are, like physiological aging, increasingly associated with pronounced disturbances in the metabolism of bioactive sphingolipids (generally tending to augment the pro-apoptotic ceramide signaling at the expense of survival signals mediated by S1P). However, recent findings suggest a more complex picture, creating the need for refinement of current knowledge on of the roles of S1P and ceramides in the various cell death modes. The early appearance of sphingolipid alterations suggests their engagement in upstream steps of disease development. These observations raise hopes for identification of therapeutic targets that would allow reaching beyond the current symptomatic treatments. They also should help in the identification of highly usable biomarkers for the still elusive goal of early diagnosis.

Besides cell survival/death signaling, the roles of sphingolipids are more obscure. Their significance in the metabolism of AβPP/Aβ and ASN, with both proteins’ physiological roles still unclear, needs extensive insights before conclusions can be drawn. Similar is the significance of sphingolipids’ links with secretion mechanisms which can affect the spread of aggregating proteins, death/survival signals, or metabolic regulators such as noncoding RNAs. The significance of miRNAs for neurodegenerative disorders, although gaining increasing recognition in the field, is still largely uncharacterized.

A final question is that about the availability of therapeutic tools to manipulate the extremely complex network of sphingolipid metabolism. Some of the most basic needs may be met with currently available repurposed drugs such as fingolimod; however, it is highly possible that exploitation of sphingolipids as therapeutic targets (as opposed to their use in diagnosis) may require significant expansion of the current toolset.
